# Sexual practices have a significant impact on the vaginal microbiota of women who have sex with women

**DOI:** 10.1038/s41598-019-55929-7

**Published:** 2019-12-24

**Authors:** Erica L. Plummer, Lenka A. Vodstrcil, Christopher K. Fairley, Sepehr N. Tabrizi, Suzanne M. Garland, Matthew G. Law, Jane S. Hocking, Katherine A. Fethers, Dieter M. Bulach, Gerald L. Murray, Catriona S. Bradshaw

**Affiliations:** 1 Central Clinical School, Monash University, The Alfred Centre, Melbourne, Victoria Australia; 2 Melbourne Sexual Health Centre, Alfred Health, Carlton, Victoria Australia; 3 Women’s Centre for Infectious Diseases, The Royal Women’s Hospital, Parkville, Victoria Australia; 4 Murdoch Children’s Research Institute, Parkville, Victoria Australia; 5 Department of Obstetrics and Gynaecology, The University of Melbourne, Parkville, Victoria Australia; 6 Kirby Institute, UNSW Australia, Kensington, NSW Australia; 7 Melbourne School of Population and Global Health, The University of Melbourne, Parkville, Victoria Australia; 8 Microbiological Diagnostic Unit Public Health Laboratory, The Peter Doherty Institute for Infection and Immunity, The University of Melbourne, Parkville, VIC Australia; 9 Melbourne Bioinformatics, The University of Melbourne, Carlton, Victoria Australia

**Keywords:** Microbiome, Bacterial infection, Epidemiology

## Abstract

Women-who-have-sex-with-women (WSW) are at increased risk of bacterial vaginosis (BV). We investigated the impact of practices and past BV on the vaginal microbiota within a two-year longitudinal cohort of Australian WSW. Self-collected vaginal swabs were used to characterise the vaginal microbiota using 16S-rRNA gene sequencing. Hierarchical clustering defined community state types (CSTs). Bacterial diversity was calculated using the Shannon diversity index and instability of the vaginal microbiota was assessed by change of CST and Bray-Curtis dissimilarity. Sex with a new partner increased the bacterial diversity (adjusted-coefficient = 0.41, 95%CI: 0.21,0.60, p < 0.001) and instability of the vaginal microbiota, in terms of both change of CST (adjusted-odds-ratio = 2.65, 95%CI: 1.34,5.22, p = 0.005) and increased Bray-Curtis dissimilarity (adjusted-coefficient = 0.21, 95%CI: 0.11,0.31, p < 0.001). Women reporting sex with a new partner were more likely than women reporting no new partner to have a vaginal microbiota characterised by *Gardnerella vaginalis* (adjusted-relative-risk-ratio[aRRR] = 3.45, 95%CI: 1.42,8.41, p = 0.006) or anaerobic BV-associated bacteria (aRRR = 3.62, 95%CI: 1.43,9.14, p = 0.007) relative to a *Lactobacillus crispatus* dominated microbiota. Sex with a new partner altered the vaginal microbiota of WSW by increasing the diversity and abundance of BV-associated bacteria. These findings highlight the influence of practices on the development of a non-optimal vaginal microbiota and provide microbiological support for the sexual exchange of bacteria between women.

## Introduction

The vaginal microbiota has an important role in protecting against a range of adverse obstetric and gynaecological outcomes including miscarriage, preterm birth, and transmission and acquisition of sexually transmitted diseases (STIs) and HIV^1–4^. The optimal vaginal microbiota of reproductive aged women is typically characterised by low bacterial diversity and high relative abundance of *Lactobacillus* spp., commonly *Lactobacillus crispatus*^5–7^.

Bacterial vaginosis (BV) is the most common vaginal dysbiosis and is characterised by a decrease in lactobacilli and increase in the diversity and abundance of facultative and strict anaerobic bacteria including *Gardnerella vaginalis*^5,8,9^. The pathogenesis of BV is complex and mounting epidemiological and microbiological evidence suggests that sexual activity has a role in both BV incidence and recurrence. Inconsistent condom use and new or increased numbers of sexual partners have been shown by meta-analysis to increase BV risk^10^. Other sexual practices associated with increased risk of BV include penile-vaginal sex^11,12^, vaginal sex after anal sex^12^, receptive oral sex with a female partner^13,14^, and shared use of sex toys between women^13,15,16^. BV prevalence is high amongst women who have sex with women (WSW) with estimates ranging from 25–52%^16–21^. Whether increased prevalence of BV in WSW is due to sexual practices or other factors is not known.

A number of studies have found sexual activity is associated with disturbance of the vaginal microbiota^22–25^, however there are limited data describing how specific sexual practices influence the vaginal microbiota in WSW. Mitchell *et al*.^26^ used culture methods and found that sharing of sex toys with female partners was associated with reduced concentration of *Lactobacillus*, and digital vaginal sex and sex toy use was associated with increased colonization of *G. vaginalis*.

Understanding how specific sexual practices influence the composition of the vaginal microbiota and contribute to vaginal dysbiosis and BV is important in order to develop effective treatment and prevention strategies. The primary objective of our study was to describe the impact of sexual practices on the vaginal microbiota of a subset of women participating in a cohort of Australian WSW.

## Results

### Description of participants at baseline and longitudinally

Baseline characteristics and sexual practices of participants are summarised in Table 1. Specimens from 102 women were initially selected for inclusion in the study; however two were removed post quality control of sequencing data (as described below), leaving 100 women in the study population. The median age of participants at enrolment was 28 years (interquartile range[IQR] 24–37 years). Most women were Australian born (86%), had tertiary level education (81%) and had a female sexual partner (FSP) at enrolment (72%). Twenty-two percent of women reported a past history of BV.

**Table 1:** Characteristics of study participants at baseline.

Characteristic	Total (N = 100)
Age^a^	
≤28	52
>28	48
Country of Birth^b^	
Australia	86
Other	14
Self-reported past history of BV	
No	78
Yes	22
Douching (ever)^c^	
No	79
Yes	20
***Baseline sexual practices***	
Current regular FSP	
No^d^	28
Yes	72
Number of FSPs in previous 12 months^a^	
≤1	60
>1	40
Ever had vaginal sex with a man	
No	26
Yes^e^	74
***Community State Type (CST) at baseline***	
CST1-*L. crispatus*	41
CST2-*Lactobacillus* mixed	19
CST3-*L. iners*	30
CST4-*G. vaginalis* and diverse	3
CST5- anaerobic and diverse^f^	7

Abbreviations: BV, bacterial vaginosis; FSP, female sexual partner;

^a^Continuous variables dichotomised at median value.

^b^n = 81 women reported Australian or English ethnicity, n = 11 reported a European ethnicity, n = 8 reported a non-European ethnicity (other ethnicities reported were Chinese, Malaysian/Indian, Indian, Israeli, Chilean).

^c^Data missing from one participant.

^d^n = 6 women reported a current MSP (male sexual partner) at baseline.

^e^n = 47 women reported ≥ 1 MSP in previous 12 months.

^f^The top five most prevalent taxa in CST5: *Dialister* spp., *Prevotella* spp., *G. vaginalis*, *L. iners* and *Peptoniphilus* spp.

Longitudinally, most women reported receiving oral sex from an FSP (85%) and use of sex toys with an FSP (72%). Fourteen women (14%) reported vaginal sex with a male during the study period. Forty women (40%) reported sex with a new partner in one or more interval (25 women reported one new sexual partner and 15 women two or more new partners over the study period). New partners were predominantly female; 28 women reported a female new partner/s, three women reported having a male new partner/s and nine women reported both female and male new partners.

A total of 372 specimens from 102 women underwent sequencing and 5,061,171 sequence reads were generated. Following quality control, 4,942,634 reads representing 393 ASVs remained. Nine specimens had <1000 reads and were excluded; consequently two participants were excluded from analysis as one did not have an enrolment specimen and one did not have longitudinal specimens post quality control. Thus, a total of 360 specimens from 100 women were included in analyses. This included 100 enrolment specimens and 260 longitudinal specimens, 47 of which represented incident BV. The median number of reads per specimen was 12,504 (IQR 7,460–18,344).

### Vaginal community state types

**Figure 1:**
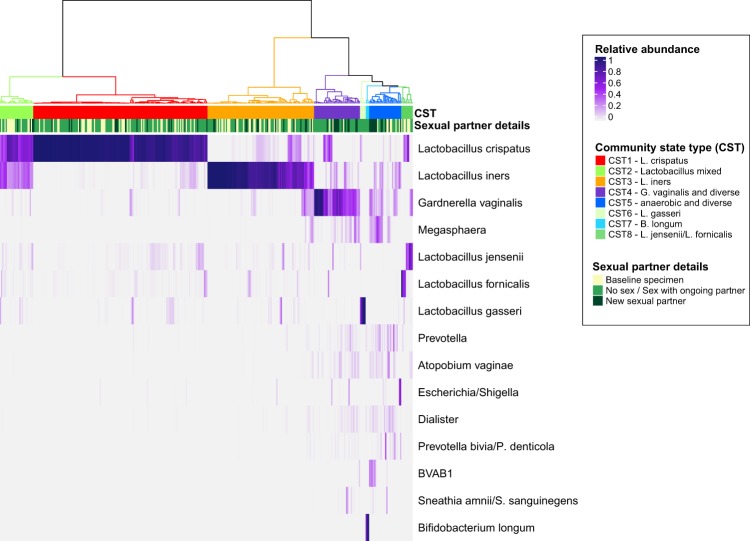
The vaginal microbiota of women who have sex with women. The heatmap displays the relative abundance of the 15 most abundant bacteria detected in women in this study. Hierarchical clustering of Euclidean distances with Ward linkage was used to determine eight community state types (CST): CST1-*L. crispatus*, CST2-*Lactobacillus* mixed, and CST3-*L. iners* (n = 93), CST4-*G. vaginalis* and diverse, CST5-anaerobic and diverse, CST6-*L. gasseri*, CST7-*Bifidobacterium longum*, CST8-*L. jensenii/L. fornicalis*. Exposure to a new sexual partner in the preceding 90 days is indicated above the heatmap.

Hierarchical clustering identified eight community state types (CSTs), Fig. 1. Five CSTs were characterised by *Lactobacillus*: CST1-*L. crispatus* (n = 152 specimens), CST2-*Lactobacillus* mixed (comprised of *L. crispatus* and *L. iners;* n = 29), and CST3-*L. iners* (n = 93), CST6-*L. gasseri* (n = 5), CST8-*L. jensenii/L. fornicalis* (n = 10). The remaining three CSTs were: CST4-*G. vaginalis* and diverse (n = 40 specimens), CST5-anaerobic and diverse (n = 28), CST7-*Bifidobacterium longum* (n = 3). The five most prevalent taxa identified in specimens in CST5 were BV-associated bacteria *Dialister* spp., *Prevotella* spp., *G. vaginalis*, *L. iners* and *Peptoniphilus* spp. For analysis purposes, the two small *Lactobacillus* CSTs (CST6 and CST8) were combined with CST2-*Lactobacillus* mixed, and CST7-*B. longum* was combined with other anaerobic dominated specimens in CST5-anaerobic and diverse.

All women at baseline had normal (Nugent Score [NS] = 0–3, n = 92/100) or intermediate microbiota (NS = 4–6, n = 8/100) by the NS method^9^. Most women (n = 90) clustered into a *Lactobacillus* dominated CST (CST1-*L. crispatus* (n = 41), CST2-*Lactobacillus* mixed (n = 19) and CST3-*L. iners* (n = 30)).

Of the longitudinal specimens with normal (NS = 0–3, n = 204) and intermediate (NS = 4–6, n = 9) microbiota, most (89%) clustered to a *Lactobacillus* CST [CST1-*L. crispatus* (n = 111/213, 52%), CST2-*Lactobacillus* mixed (n = 24/213; 11%) and CST3-*L. iners* (n = 55/213; 26%)]. The majority of incident BV specimens (NS = 7–10, n = 47) clustered with CST4-*G. vaginalis* and diverse (n = 26/47; 55%) and CST5-anaerobic and diverse (n = 12/47; 26%).

### Factors associated with vaginal microbiota diversity

In univariate analyses, sex with a new partner compared with no sex or sex in an ongoing relationship (defined as relationship for >3 months) was significantly associated with increased bacterial diversity of the vaginal microbiota (coefficient = 0.49, 95%CI: 0.30,0.68, p < 0.001; Table 2). Other characteristics associated with increased microbiota diversity included self-reported past history of BV, smoking, having two or more sexual partners in a study interval (i.e. the period of time between two specimen collections), frequent sexual activity (several times/week), receptive oral sex from any FSP and self-report of BV symptoms (abnormal vaginal odour and/or vaginal discharge; Table 2). Douching and sharing of sex toys had a borderline association with increased diversity.

**Table 2:** Characteristics and sexual practices associated with bacterial diversity of the vaginal microbiota.

Characteristic	n women reporting exposure, n intervals exposure reported (N = 100 women and 360 intervals)	Coeff. (95% CI)^a^	*P value*^*a*^	Adj Coeff. (95% CI)^b^	*P value*^*b*^
Self-reported past history of BV					
No	78, 285	ref		ref	
Yes	22, 75	0.28 (0.06,0.50)	**0.013**	0.26 (0.04,0.48)	**0.018**
***Longitudinal characteristics***^***c***^					
Any smoking^d,e^					
No	47, 210	ref		ref	
Yes	53, 149	0.18 (0.01,0.35)	**0.036**	0.08 (−0.09,0.25)	0.352
Any douching^e^					
No	94, 351	ref			
Yes	6, 8	0.50 (−0.02,1.01)	0.060		
Number of SP					
0	3, 38^f^	ref			
1	67, 270^f^	0.03 (−0.23,0.29)	0.818		
≥2	30, 52^f^	0.36 (0.04,0.67)	**0.027**		
Frequency of sex					
Once/month or less	14, 118^g^	ref		ref	
Several times/month	36, 131^g^	0.17 (−0.02,0.36)	0.074	0.13 (−0.05,0.31)	0.164
Several times/week	50, 111^g^	0.30 (0.10,0.51)	**0.003**	0.20 (0.00,0.41)	**0.049**
Sex with NP^h^					
No	60, 300	ref		ref	
Yes	40, 60	0.49 (0.30,0.68)	**<0.001**	0.41 (0.21,0.60)	**<0.001**
***Sexual practices with an FSP***^***i***^					
Any receptive oral vaginal sex					
No^j,e^	15, 138	ref		ref	
Yes	85, 221	0.24 (0.08,0.40)	**0.003**	0.15 (−0.02,0.31)	0.076
Any digital anal sex					
No^j,e^	77, 317	ref			
Yes	23, 41	0.32 (0.08,0.57)	**0.010**		
Sharing of sex toys^e^					
No toys/washed/condoms used^j^	58, 272	ref			
Unwashed	42, 87	0.18 (−0.00,0.37)	0.051		
***Sexual practices with an MSP***^k^					
Any vaginal sex					
No^l, e^	86, 321	ref			
Yes	14, 38	0.07 (−0.21,0.35)	0.637		
***Self-reported symptoms***					
Self-reported abnormal vaginal discharge and/or odour					
No	72, 317	ref			
Yes	28, 43	0.32 (0.09,0.55)	**0.007**		

Abbreviations: BV, bacterial vaginosis; SP, sexual partner (may refer to FSP or MSP); NP, new partner (may refer to FSP or MSP); FSP, female sexual partner; MSP, male sexual partner

Missing data for variables included in this analysis occurred in <0.5% of intervals.

^a^Univariate GEE linear regression, where participant ID is panel variable. The regression coefficient represents the mean difference of Shannon diversity between the reference and comparison group/s for each characteristic/practice investigated.

^b^Multivariable GEE linear regression, where participant ID is panel variable.

^c^Longitudinal characteristics were measured as any exposure over the prior study interval (~90 days). No significant associations were identified between Shannon diversity and either hormonal contraceptive use or recent menses.

^d^There was no dose-response relationship between smoking and Shannon diversity.

^e^Missing data from a maximum of two intervals for some variables.

^f^For women reporting different numbers of sexual partners in two or more intervals, the category representing the highest number of sexual partners has been used to calculate n women reporting exposure.

^g^For women reporting different frequencies of sexual activity in two or more intervals, the most frequent category has been used to calculate n women reporting exposure.

^h^Sex with a new partner with who first sexual contact was within 90 days.

^i^No significant associations were identified between Shannon diversity and the following sexual practices with an FSP: Any digital vaginal sex from an FSP, any receptive oral anal sex from an FSP and current FSP with BV symptoms. These practices have been left out to simplify the table.

^j^Or did not have a FSP.

^k^No significant associations were identified between Shannon diversity and the following sexual practices with an MSP: Condoms used for vaginal sex, vaginal sex after anal sex, any receptive oral vaginal sex from an MSP, any digital vaginal sex from an MSP, any anal sex from an MSP. These practices have been left out to simplify the table.

^l^Or did not have a MSP.

We included sex with a new partner, frequency of sex, smoking, oral sex and past history of BV in a multivariable model (Table 2). Digital anal sex was not included in adjusted analyses to limit over-fitting the model. Sex with a new partner (adjusted coefficient = 0.41, 95%CI: 0.21,0.60, p < 0.001), frequent sex (adj. coefficient = 0.20, 95%CI: 0.00,0.41, p = 0.049) and past history of BV (adj. coefficient = 0.26, 95%CI: 0.04,0.48, p = 0.018) were significantly associated with increased diversity of the vaginal microbiota. Smoking and receptive oral sex with an FSP were not associated with diversity adjusted analyses.

To explore the relationship between oral sex, exposure to a new sexual partner, and microbiota diversity, we investigated 1) the impact of new partner exposure on diversity stratified by receptive oral sex, and 2) investigated the interaction between new partner exposure and receptive oral sex. Although new partner exposure was significantly associated with microbiota diversity in women reporting oral sex and not in women who did not practice oral sex (Supplementary Table 1), the 95% confidence intervals overlapped, suggesting no statistical difference in the effect of new partner by oral sex, and furthermore there was no evidence of interaction when formally tested (p = 0.110).

### Factors associated with instability of the vaginal microbiota

Compositional change (instability) was measured by change of CST and Bray-Curtis dissimilarity score, calculated between consecutive longitudinal specimens.

Eighty-three women (83%) experienced at least one change of CST during the study period, accounting for 138 instances of CST change. Interestingly, changing between different *Lactobacillus* CSTs (n = 66/138, 48% of all CST changes) was as common as changing from a *Lactobacillus* CST to CST4-*G. vaginalis* and diverse or CST5-anaerobic and diverse (n = 50/138, 36%).

Practices significantly associated with change of CST by univariate analysis (smoking and sex with a new partner) were included in a multivariable model with CST of index specimen (i.e. the first specimen of each consecutive pair; Table 3). In the adjusted analysis, sex with a new partner (adjusted odds ratio [AOR] 2.65, 95%CI: 1.34,5.22, p = 0.005) and smoking (AOR 1.79, 95%CI: 1.03,3.11, p = 0.039) were both associated with an increased odds of change of CST when adjusted for CST of index specimen. Additionally, women with a vaginal microbiota classified as CST2-*Lactobacillus* mixed (AOR = 6.65, 95%CI: 2.81,15.76, p < 0.001), CST3-*L. iners* (AOR = 3.13, 95%CI: 1.67,5.87, p < 0.001) or CST5-anaerobic and diverse (AOR 13.18, 95%CI: 2.83,61.31, p < 0.001) were more likely to change CST in the next interval compared with women with a vaginal microbiota of CST1-*L. crispatus*. Having a CST4-*G. vaginalis* and diverse microbiota was not significantly associated with change of CST, likely because the majority of CST4 samples represented an endpoint specimen i.e. incident BV (n = 26/40, 65%).

**Table 3:** Characteristics and sexual practices associated with instability of the vaginal microbiota as measured by change of CST between consecutive specimens.

Characteristic	OR 95% CI	*P value*^*a*^	AOR 95% CI	*P value*^*b*^
Self-reported past history of BV				
No	1			
Yes	1.21 (0.64,2.29)	0.553		
***Longitudinal practices***^***cd***^				
Any smoking^e^				
No	1		1	
Yes	2.10 (1.25,3.54)	**0.005**	1.79 (1.03,3.11)	**0.039**
No. of cigarettes smoked				
0/non-smoker	1			
1–7	1.98 (0.87,4.49)	0.104		
8+	1.61 (0.78,3.33)	0.198		
Number of SP				
0	1			
1	0.74 (0.33,1.67)	0.464		
≥2	2.12 (0.73,6.14)	0.165		
Frequency of sex				
Once/month or less	1			
Several times/month	0.98 (0.54,1.77)	0.948		
Several times/week	1.59 (0.84,3.11)	0.152		
Sex with NP^e^				
No	1		1	
Yes	2.56 (1.37,4.81)	**0.003**	2.65 (1.34,5.22)	**0.005**
***Sexual practices with FSP***^***f***^				
Any receptive oral vaginal sex				
No^g^	1			
Yes	1.59 (0.96,2.66)	0.074		
Sharing of sex toys				
No toys/washed/condoms used^g^	1			
Unwashed	1.50 (0.83,2.72)	0.182		
***Sexual Practices with an MSP***^***h***^				
Any vaginal sex				
No^i^	1			
Yes	0.72 (0.32,1.63)	0.435		
***Self-reported symptoms and microbiota characteristics***				
Self-reported abnormal vaginal discharge and/or odour				
No	1			
Yes	1.04 (0.49,2.22)	0.917		
Shannon diversity	1.53 (1.02,2.28)	**0.038**		
Community state type (CST) of index specimen^j^				
CSTI- *L. crispatus*	1		1	
CST2-*Lactobacillus* mixed	5.71 (2.47,13.15)	**<0.001**	6.65 (2.81,15.76)	**<0.001**
CST3-*L. iners*	2.81 (1.54,5.12)	**0.001**	3.13 (1.67,5.87)	**<0.001**
CST4-*G. vaginalis* and diverse^k^	1.57 (0.50,4.95)	0.440	1.57 (0.48,5.17)	0.457
CST5- anaerobic and diverse	14.22 (3.13,64.69)	**<0.001**	13.18 (2.83,61.31)	**<0.001**

Abbreviations: BV, bacterial vaginosis; SP, sexual partner (may refer to female or male partner); NP, new partner (may refer to FSP or MSP); FSP, female sexual partner; MSP, male sexual partner.

Missing data for variables included in this analysis occurred in <0.5% of intervals.

^a^Univariate GEE logistic regression clustered for multiple specimens from each participant.

^b^Multivariable GEE logistic regression clustered for multiple specimens from each participant.

^c^Longitudinal characteristics were measured as any exposure over the prior study interval (~90 days). No significant associations were identified between change of CST and either hormonal contraceptive use or recent menses.

^d^Douching omitted from table due to collinearity – all intervals of douching (n = 5) were accompanied by a change of CST.

^e^Sex with a new partner with who first sexual contact was within 90 days.

^f^The following sexual practices/characteristics with an FSP were left out of the table for simplicity: digital vaginal sex, receptive oral anal sex, digital anal sex, and current partner with BV symptoms. No significant associations between change of CST and these sexual practices were identified.

^g^Or did not have a FSP.

^h^The following sexual practices/characteristics with an MSP were left out of the table for simplicity: condoms use for vaginal sex, anal sex, vaginal sex after anal sex, oral vaginal sex and digital vaginal sex. No significant associations between change of CST and these sexual practices were identified.

^i^Or did not have a MSP.

^j^Index specimen refers to the first specimen of each consecutive pair.

^k^Majority of CST4 specimens are endpoint specimens which do not have accompanying change of CST information.

**Figure 2:**
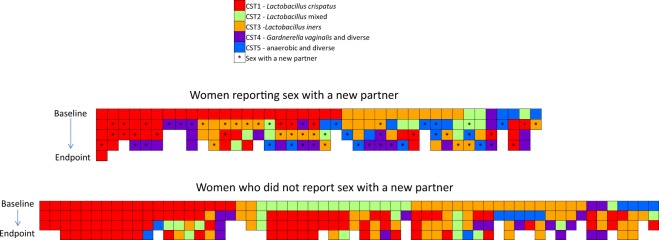
Longitudinal changes of community state type in women reporting sex with a new partner. Each column represents a participant and sequential longitudinal specimens are ordered from baseline to endpoint. Boxes are coloured according to community state type (CST). *Indicates a report of sex with a new partner. Most women changed CST at least once during the study. Change of CST occurred in 36/40 (90%) women who reported exposure to a new partner and 47/60 (78%) women who did not report sex with a new partner.

By multinomial regression, women reporting sex with a new partner were more likely than women without a new partner to change from a *Lactobacillus* CST (i.e. CST1/2/3) to a non-*Lactobacillus* dominated CST relative to not changing CST (relative risk ratio [RRR] = 4.18, 95%CI: 2.06,8.50, p < 0.001). Smokers were more likely than non-smokers to change between *Lactobacillus* CSTs (RRR = 2.21, 95%CI: 1.15,4.23, p = 0.017) or change from a *Lactobacillus* CST to a non-*Lactobacillus* dominated CST (i.e. CST4/5; RRR = 2.04, 95%CI: 1.11,3.75, p = 0.021) relative to not changing CST. Figure 2 summarises changes of CST in each participant longitudinally and indicates when sex with a new partner was reported.

Practices significantly associated with instability of the vaginal microbiota (i.e. increased Bray-Curtis scores between consecutive samples) by univariate analysis were included in a multivariable model that also included CST of the index specimen (Table 4). Sex with a new partner (adj. coefficient = 0.21, 95%CI: 0.11, 0.31, p < 0.001) and smoking (adj. coefficient = 0.09, 95%CI: 0.01, 0.18, p = 0.036) were associated with increased instability of the microbiota, adjusted for index specimen CST. Additionally, having a vaginal microbiota in the index specimen of CST3-*L. iners* (adj. coefficient = 0.25, 95%CI: 0.15,0.34, p < 0.001), CST4-*G. vaginalis* and diverse (adj. coefficient = 0.24, 95%CI: 0.05,0.43, p = 0.013) or CST5-anaerobic and diverse (adj. coefficient = 0.44, 95%CI: 0.30,0.60, p < 0.001) was associated with increased instability of the vaginal microbiota longitudinally compared to a *L. crispatus* (CST1) vaginal microbiota.

**Table 4:** Characteristics and sexual practices associated with instability of the vaginal microbiota as measured by Bray-Curtis dissimilarity between consecutive specimens.

Characteristic	Coeff. 95% CI^a^	*P value*^a^	Adj Coeff. 95% CI^b^	*P value*^b^
Self-reported past history of BV				
No	ref	—		
Yes	0.10 (−0.04,0.23)	0.159		
***Longitudinal practices***^***c***^				
Any smoking				
No	ref	—	ref	—
Yes	0.15 (0.05,0.25)	**0.003**	0.09 (0.01,0.18)	**0.036**
No. of cigarettes smoked				
0/non-smoker	ref	—		
1–7	0.07 (−0.09,0.22)	0.388		
8+	0.15 (0.01,0.30)	**0.035**		
Any douching				
No	ref	—		
Yes	0.33 (−0.01,0.67)	0.056		
Number of SP				
0	ref			
1	−0.12 (−0.28,0.03)	0.119		
≥2	0.13 (−0.06,0.32)	0.182		
Frequency of sex				
Once/month or less	ref	—		
Several times/month	−0.01 (−0.12,0.10)	0.865		
Several times/week	0.06 (−0.07,0.18)	0.366		
Sex with NP^d^				
No	ref	—	ref	—
Yes	0.23 (0.12,0.33)	**<0.001**	0.21 (0.11,0.31)	**<0.001**
***Sexual practices with FSP***^**e**^				
Any receptive oral vaginal sex				
No^f^	ref	—		
Yes	0.06 (−0.04,0.15)	0.258		
Sharing of sex toys				
No toys/washed/condoms used^f^	ref			
Unwashed	0.04 (−0.07,0.15)	0.478		
***Sexual practices with MSP***^***g***^				
Any vaginal sex				
No^h^	ref	—		
Yes	0.09 (−0.07,0.26)	0.279		
***Self-reported symptoms and microbiota characteristics***				
Self-reported abnormal vaginal discharge and/or odour				
No	ref	—		
Yes	0.07 (−0.07,0.22)	0.305		
Shannon diversity	0.07 (−0.00,0.14)	0.053		
Community state type (CST) of index specimen^i^				
CSTI-*L. crispatus*	ref	—	ref	—
CST2-*Lactobacillus* mixed	0.08 (−0.05,0.20)	0.241	0.09 (−0.03,0.22)	0.134
CST3-*L. iners*	0.23 (0.12,0.33)	**<0.001**	0.25 (0.15,0.34)	**<0.001**
CST4-*G. vaginalis* and diverse^j^	0.23 (0.03,0.43)	**0.027**	0.24 (0.05,0.43)	**0.013**
CST5- anaerobic and diverse	0.47 (0.30,0.64)	**<0.001**	0.44 (0.30,0.60)	**<0.001**

Abbreviations: BV, bacterial vaginosis; SP, sexual partner (may refer to FSP or MSP); NP, new partner (may refer to FSP or MSP); FSP, female sexual partner; MSP, male sexual partner.

Missing data for variables included in this analysis occurred in <0.5% of intervals.

^a^Univariate GEE linear regression clustered for multiple specimens from each participant. The regression coefficient represents the mean difference of Bray-Curtis Dissimilarity between the reference and comparison group/s for each characteristic/practice investigated.

^b^Multivariable GEE linear regression clustered for multiple specimens from each participant.

^c^Longitudinal characteristics were measured as any exposure over the prior follow-up interval (~90 days). No significant associations were identified between beta diversity and either hormonal contraceptive use or recent menses.

^d^Sex with a new partner with who first sexual contact was within 90 days. Partner gender was defined by the participant.

^e^The following sexual practices/characteristics with an FSP were left out of the table for simplicity: digital vaginal sex, receptive oral anal sex, digital anal sex and current partner with BV symptoms. No significant associations between beta diversity and these sexual practices were identified.

^f^Or did not have a FSP.

^g^The following sexual practices/characteristics with an MSP were left out of the table for simplicity: condoms use for vaginal sex, anal sex, vaginal sex after anal sex, oral vaginal sex and digital vaginal sex. No significant associations between beta diversity and these sexual practices were identified.

^h^Or did not have a MSP.

^i^Index specimen refers to the first specimen of each consecutive pair.

^j^Majority of CST4 specimens are endpoint specimens which do not have accompanying beta diversity information.

### Practices impacting the vaginal microbiota composition

After considering factors that influence stability of the microbiota, we looked at specific characteristics and sexual practices that influenced the vaginal microbiota composition by multinomial logistic regression. In univariate analyses (Supplementary Table 2), we found women who reported sex with a new partner in the previous 90 days were more likely than women reporting no sex or sex in an ongoing relationship to have a vaginal microbiota of CST4-*G. vaginalis* abundant and diverse (RRR = 4.09, 95%CI: 1.69,9.92, p = 0.002) or CST5-anaerobic and diverse (RRR = 5.37, 95%CI: 2.18,13.20, p < 0.001) than one of CST1. Women who reported smoking were more likely than non-smokers to have anaerobic microbiota (CST5) relative to CST1 (RRR = 3.01,95%CI: 1.31,6.92, p = 0.009). Women who reported receptive oral vaginal sex from an FSP or sharing of unwashed sex toys with an FSP were more likely to have a CST4 microbiota, and women who douched or had a past history of BV were more likely to have a CST5 microbiota (Supplementary Table 2). Women reporting recent menses (defined as onset of menses within 7 days of specimen collection) were more likely than women not reporting recent menses (>7 days from specimen collection) to have a CST2-*Lactobacillus*-mixed or CST3-*L. iners* microbiota composition relative to CST1 microbiota, but were not more likely to have a *G. vaginalis* (CST4) or anaerobic microbiota (CST5).

We included past history of BV, receptive oral sex from a FSP, sex with a new partner, sharing of unwashed sex toys with an FSP smoking and recent menses in a multivariable multinomial regression model (Table 5). Women reporting sex with a new partner were more likely than women reporting no sex or sex in an ongoing relationship to have a CST4-*G. vaginalis* and diverse (adjusted-RRR = 3.45, 95%CI: 1.42,8.41, p = 0.006) or CST5-anerobic and diverse vaginal microbiota (adjusted-RRR = 3.62, 95%CI: 1.43,9.14, p = 0.007) relative to a CST1 vaginal microbiota. Women reporting that they shared unwashed sex toys with an FSP were more likely than women not reporting this practice to have a CST4 vaginal microbiota (adjusted-RRR = 2.49, 95%CI: 1.05,5.91, p = 0.038). Women reporting smoking were more likely than non-smokers to have a CST5-anaerobic and diverse vaginal microbiota relative to a CST1 vaginal microbiota (adjusted-RRR = 2.94, 95%CI: 1.16,7.43, p = 0.023). Women with a past-history of BV were more likely to have a CST5 vaginal microbiota (adjusted-RRR = 3.18, 95%CI: 1.13,8.91, p = 0.028), and women reporting recent menses were more likely to have a CST2 (adjusted-RRR = 3.89, 95%CI: 1.58,9.50, p = 0.003) or CST3 (adjusted-RRR = 2.37, 95%CI: 1.14,4.90, p = 0.020) vaginal microbiota.

**Table 5:** Characteristics and practices associated with vaginal microbiota composition by multivariable multinomial logistic regression.

Outcome by CST	RRR (95% CI)	*P value*^a^	Adjusted RRR (95% CI)	*P value*^b^
***Lactobacillus*** **mixed (CST2) vs CST1**				
Self-reported past history of BV^c^	0.87 (0.35,2.17)	0.762	0.83 (0.33,2.09)	0.696
Smoker^d^	1.59 (0.77,3.29)	0.214	1.75 (0.84,3.65)	0.138
Sex with a NP^e^	0.62 (0.18,2.19)	0.460	0.57 (0.16,2.00)	0.384
Receptive oral vaginal sex from FSP^f^	1.09 (0.56,2.15)	0.799	1.02 (0.51,2.05)	0.951
Sharing of unwashed sex toys with FSP^g^	0.92 (0.36,2.32)	0.859	0.75 (0.26,2.18)	0.600
Onset of last menses ≤7 days ago^h^	3.59 (1.49,8.68)	**0.004**	3.89 (1.58,9.50)	**0.003**
***L. iners*** **(CST3) vs CST1**				
Self-reported past history of BV^c^	0.69 (0.26,1.87)	0.467	0.66 (0.23,1.93)	0.450
Smoker^d^	1.38 (0.71,2.65)	0.341	1.46 (0.72,2.94)	0.291
Sex with a NP^e^	1.77 (0.87,3.60)	0.117	1.61 (0.77,3.38)	0.208
Receptive oral vaginal sex from FSP^f^	1.09 (0.57,2.12)	0.790	1.01 (0.51,2.01)	0.977
Sharing of unwashed sex toys with FSP^g^	0.98 (0.50,1.92)	0.952	1.02 (0.48,2.14)	0.965
Onset of last menses ≤7 days ago^h^	2.19 (1.08,4.46)	**0.030**	2.37 (1.14,4.90)	**0.020**
***G. vaginalis*** **and diverse (CST4) vs CST1**				
Self-reported past history of BV^c^	1.13 (0.39,3.27)	0.817	1.24 (0.43,3.57)	0.686
Smoker^d^	1.72 (0.74, 4.01)	0.207	1.69 (0.70,4.08)	0.240
Sex with a NP^e^	4.09 (1.69,9.92)	**0.002**	3.45 (1.42,8.41)	**0.006**
Receptive oral vaginal sex from FSP^f^	2.60 (1.09,6.20)	**0.031**	1.94 (0.77,4.84)	0.158
Sharing of unwashed sex toys with FSP^g^	2.38 (1.04,5.45)	**0.039**	2.49 (1.05,5.91)	**0.038**
Onset of last menses ≤7 days ago^h^	1.64 (0.55,4.91)	0.378	1.37 (0.41,4.64)	0.611
**Anaerobic and diverse (CST5) vs CST1**				
Self-reported past history of BV^c^	2.82 (1.09,2.27)	**0.032**	3.18 (1.13,8.91)	**0.028**
Smoker^d^	3.01 (1.31,6.92)	**0.009**	2.94 (1.16,7.43)	**0.023**
Sex with a NP^e^	5.37 (2.18,13.20)	**<0.001**	3.62 (1.43,9.14)	**0.007**
Receptive oral vaginal sex from FSP^f^	2.17 (0.86,5.46)	0.099	1.67 (0.62,4.49)	0.308
Sharing of unwashed sex toys with FSP^g^	1.46 (0.62,3.46)	0.387	2.07 (0.79,5.43)	0.141
Onset of last menses ≤7 days ago^h^	1.03 (0.27,4.00)	0.964	0.78 (0.01,0.12)	0.734

Abbreviations: CST, community state type; NP, new partner (may refer to FSP or MSP); FSP, female sexual partner.

Missing data for variables included in this analysis occurred in <0.5% of intervals.

^a^Multinomial logistic regression with CST1-*L. crispatus* as the baseline comparison group. Analysis clustered for multiple specimens from participants (100 clusters).

^b^Multinomial logistic regression as described in a adjusted for all other characteristics in the table.

^c^Self-reported past history of BV relative to no self-report of past history of BV.

^d^Smoker relative to non-smoker.

^e^Sex with a new partner with who first sexual contact was within 90 days relative to no sex/sex with a partner with who first sexual contact was >90 days.

^f^Receptive oral vaginal sex from FSP relative to no receptive oral sex from FSP (or no FSP).

^g^Sharing of unwashed sex toys with FSP relative to no toy use/changed condoms on the sex toys/always washed the sex toys between sharing with a partner.

^h^Onset of last menses ≤7 days ago relative to onset of last menses >7 days ago.

## Discussion

In this longitudinal cohort study of women who have sex with women, specific sexual practices influenced the bacterial diversity, stability and composition of the vaginal microbiota. Sex with a new partner (primarily representing new FSPs) was associated with an increase in bacterial diversity and an increase in compositional change (or instability) of the vaginal microbiota, both in terms of change of CST and increased Bray-Curtis dissimilarity. Furthermore, women who reported sex with a new partner were more likely than women reporting no sex/sex in an ongoing relationship to have a vaginal microbiota characterised by BV-associated anaerobic bacteria or *G. vaginalis*, relative to an optimal microbiota characterised by *L. crispatus*. This study highlights the influence of practices on the development of a non-optimal vaginal microbiota and provides microbiological support for the sexual exchange of bacteria between women. These microbiological findings complement the previously reported epidemiological data from the original cohort^13,18^ which showed sex with a new partner was associated with a 2.5-fold increased risk of BV acquisition.

There is increasing evidence to support the sexual transmission of vaginal bacteria between WSW. Longitudinal studies in this population have shown that one of the greatest risk factors for BV is having a sexual partner with a history of BV, BV symptoms or microbiologically confirmed BV^13,14^. A recent study demonstrated that incident BV occurred at a median of 4 days post sexual activity in 93% of WSW, indicating a similar incubation period to that of other STIs^27^. An early study looking at the transmission dynamics of BV demonstrated that transfer of vaginal secretions between women resulted in BV in 11 of 15 women^28^. Furthermore, high concordance of Nugent Score categories between FSP^13,15,17–19^ and evidence that women in monogamous relationships share *Lactobacillus* strains^29^ in their vaginas supports exchange of bacteria between women during sex. In our study, women who shared unwashed sex toys and/or received oral sex from an FSP were more likely than women not reporting these practices to have an anaerobic or *G. vaginalis* abundant vaginal microbiota than a microbiota dominated by *L. crispatus*. Sexual practices are frequently highly correlated, so it is difficult to determine whether one activity has a greater impact on the vaginal microbiota than others. However, both oral sex with an FSP and sex toy use involve exchange of bodily fluids to varying degrees and therefore promote exchange of bacteria between women. Additionally, both practices have been reported as a risk factor for BV^10,13,14,16^. Collectively, these data suggest that female partner treatment of women with BV may be an effective strategy to improve BV cure and warrants further investigation.

Change of CST was common in our study, in accordance with previous reports that show the vaginal microbiota can be highly dynamic^22,23,30^. Compositional change (or instability) of the vaginal microbiota between consecutive specimens was primarily influenced by the bacteria present in the index specimen. Collectively, women with a low diversity *L. crispatus* dominated vaginal microbiota were more likely to have a stable microbiota longitudinally and were less likely to experience change of CST than women with a diverse microbiota or a microbiota abundant in *L. iners* or *G. vaginalis*. Our findings are consistent with one study^22^ that analysed the vaginal microbiota of 32 women sampled twice-weekly for 16-weeks. Gajer *et al*.^22^ reported that *L. crispatus* and *L. gasseri* dominated microbiota appeared to be stable, and that sexual activity negatively impacted stability. Interestingly, practices and microbiological characteristics associated with change of CST were highly consistent with those associated with increasing instability of the microbiota (measured by Bray-Curtis), suggesting change of CST may be a useful measure of microbiota instability^31^.

Smoking had a broad ranging effect on the diversity, stability and composition of the vaginal microbiota, and past studies have shown an association between smoking and BV and/or vaginal microbiota composition that was dose dependent^18,32–34^. There are a number of possible explanations for this association. Smokers have been shown to have reduced oestradiol levels compared non-smokers^35^, and reduced oestrogen has been associated with non-optimal *Lactobacillus*-deficient vaginal microbiota^36^. Furthermore, it is well established that nicotine has detrimental effects on the immune system, including reduced production of inflammatory cytokines and decreased functionality of neutrophils and macrophages^37^, and nicotine and its derivatives have been detected in the vaginal metabolome^38^. It is possible that modulation of immune responses may result in reduced clearance of *G. vaginalis* and other BV-associated bacteria (similar to what has been observed for human papillomavirus^39^) or prevent maintenance of an optimal *Lactobacillus* vaginal microbiota. The association between smoking and vaginal microbiota instability seen in our study is interesting and may be because the microbiota composition that is found more commonly in smokers (i.e. anaerobic and diverse microbiota) is inherently more unstable than others, such as those dominated by *L. crispatus*. It is also possible that observed associations between smoking and adverse microbiota composition and instability are due to unmeasured confounding; however, the fact that this association has been shown to be dose dependent in some studies and persists in adjusted analysis provides evidence for a biological association.

A number of other factors were associated with vaginal microbiota composition, stability and/or diversity including past history of BV, menses and douching. The finding that past history of BV was associated with both increased bacterial diversity and an anaerobic microbiota may represent persistence or re-emergence of a polymicrobial BV-biofilm^40,41^, or alternatively the influence of other factors such as host genetics or immune function^37^, diet^42^ or contraceptive practices^43^. Both douching and menses have been shown in a number of studies to adversely alter vaginal microbiota composition and stability^22,44,45^, and consistent with this, we found that douching was associated with anaerobic and diverse vaginal microbiota and had a borderline adverse effect on microbiota stability in univariate analyses. While recent menses did not have an effect on microbiota diversity or stability in our study, it did influence microbiota composition. Women were more likely to have a vaginal microbiota abundant in *L. iners* (i.e. CST2 or CST3) if their specimen was collected within seven days of onset of menses, consistent with data that shows *L. iners* grows best on media containing blood^46,47^.

Hormonal contraception may have a beneficial impact on the vaginal microbiota^48^. However, we found no association between hormonal contraception and microbiota diversity, stability or composition, which may be because only a small number of women reported hormonal contraceptive use in the parent cohort.

There are a number of limitations to this study. The study population comprised highly educated women who were predominately Australian born and reported Australian or English ethnicity, which may limit the generalizability of our findings. Specimens were collected every three months which limited our ability to assess immediate effect of sexual practices behaviours on the vaginal microbiota and any short-term fluctuations in microbiota composition. Specimens included in the analysis were not selected randomly or from specified study time points which may have biased results. We did not include negative controls alongside specimens during sequencing, however we removed contaminants previously identified using the same extraction methodology, primer set up and sequencing instrument^49^ and the microbiota profiles are consistent with those previously published^6,25,50^. Finally, this study did not assess practices or the vaginal microbiota of the sexual partner/s of participants so we cannot definitively prove sexual transmission of BV-associated bacteria is occurring between women. Nevertheless, the microbiota data presented here is consistent with epidemiological data that supports sexual transmission of BV in WSW^13,14^.

This study shows that sex with a new partner is associated with changes in the vaginal microbiota of WSW, including increased diversity and increased abundance of bacteria commonly associated with a non-optimal vaginal microbiota. These findings suggest that sexual exchange of bacteria, including BV-associated bacteria, occurs between female sexual partners, and highlight the influence of specific practices on the development of a non-optimal vaginal microbiota. These data are important for informing strategies to promote a vaginal microbiota that is associated with optimal reproductive health, as well as new approaches to improve BV cure such as female partner treatment.

## Methods

### Participant and specimen selection

Participants were selected from the Women On Women’s (WOW) Health study, a two-year cohort of 298 WSW designed to examine epidemiological and microbiological factors associated with incident BV^13,18^. Women reported a FSP within 18 months prior to enrolment and were BV negative (NS < 7^9^) on three consecutive baseline vaginal smears collected one week apart. Women self-collected a vaginal swab and smear, and completed a detailed questionnaire every three months until study endpoint (diagnosis of incident BV [NS = 7–10] or 24 months without BV). Women were instructed to avoid specimen collection on the heaviest days of their menstrual cycle^13^.

For the microbiota sub-study, we included all women who developed incident BV (n = 51) and an equal number of women who did not (initially controls were over-selected using a random sort command in Stata/IC (v14.2, StataCorp LP, College Station, USA)). Seven of the 51 women to develop incident BV co-enrolled in the original cohort with their FSP^13^. As such, controls were then frequency matched on co-enrolment status and age to ensure a similar distribution of both variables (for example, the last non co-enrolled control was replaced with the next randomly selected co-enrolled control). Each woman contributed a baseline specimen and an endpoint specimen (BV-specimen from women with incident BV or the 24 month specimen from women without BV). Up to three interim specimens were included for each woman (typically the last two specimens collected prior to the endpoint specimen). If a specimen could not be used/located, an earlier specimen from that participant was used.

Ethical approval was obtained from the Human Research Ethics Committees of Alfred Hospital, Melbourne, Australia and the University of Melbourne. All research was performed in accordance with the National Statement on Ethical Conduct in Human Research. Informed written consent was obtained from all participants for the use of their specimens in the current study.

### Laboratory methods

Swabs were agitated in 1 mL RNAlater (Thermo Fisher Scientific, Waltham, USA) and stored at −80 °C prior to DNA extraction using the MagNA Pure 96 instrument and the DNA and Viral NA small volume kit (Roche Diagnostics, Mannheim, Germany). Dual index primers 341 F/805 R with heterogeneity spacers^51–53^ were used for PCR amplification of the V3-V4 hypervariable regions of the 16S rRNA gene. Libraries were sequenced by Micromon Genomics (Micromon, Monash University, Victoria, Australia) on the MiSeq platform (Illumina, San Diego, CA, USA). Sequence reads are available in the NCBI Sequence Read Archive under Bioproject PRJNA434520.

### Sequence data analysis

Barcodes were extracted using QIIME v1.9.0^54^ and demultiplexing was performed using idemp (https://github.com/yhwu/idemp). Primers and heterogeneity spacers were removed using TagCleaner standalone version 0.16^55^. Reads were processed using DADA2 v1.6.0^56^. Reads were truncated based on quality profiles (at 250 bases for read 1 and 220 bases for read 2) and were discarded if they had ambiguous bases or exceeded the number of expected errors based on quality scores. Chimeras were identified and removed. Taxonomy was assigned using the default RDP Classifier implemented in DADA2 and the Silva reference database (v128)^57^. Species level assignment was performed using exact matching in the DADA2 package and taxonomy for *Lactobacillus* spp. was confirmed by a BLAST search against a database of 16S rRNA gene sequences from 158 type strains. Not all ASVs were able to be assigned to species level.

BV-associated bacteria (BVAB)−1 has previously been misclassified as *Shuttleworthia*^58^ and BVAB3 is named as *Fastidiosiplia* in the Silva database^59^. We aligned *Shuttleworthia* and *Fastidiosiplia* ASVs against BVAB1 (NCBI GenBank AY724739.1), BVAB2 (AY724740.1) and BVAB3 (AY724741.1) using Clustal Omega (EMBL-EBI)^60,61^. *Shuttleworthia* ASV had 100% identity to BVAB1. Two *Fastidiosiplia* ASVs had high identity to BVAB2 (99.50 and 100% identity, respectively), and a third *Fastidiosiplia* ASV had 100% identity to BVAB3. The ASVs were reclassified accordingly.

ASVs were removed if they had a total abundance of less than 0.001% or were present in only one specimen. The ASV table was screened for contaminants previously identified in negative controls^49^, as well as common sequencing contaminants (removed ASVs belonging to *Facklamia* and *Shewanella* genera and Halomonadaceae family)^62,63^. Specimens with fewer than 1000 reads were excluded from analysis. Participants were excluded if they did not have an enrolment specimen or did not have any follow-up specimens.

Diversity metrics and CST were generated using the Vegan package^64^ and R Studio [V 1.1.419, Boston, USA] employing R v3.4.3. Alpha diversity was calculated using the Shannon Diversity Index using ASV data. ASVs assigned to the same taxonomy were merged and the relative abundance of each taxon was used for CST identification. Hierarchical clustering of Euclidean distances with Ward linkage was performed on the relative abundance of each taxon and a scree plot of within cluster distances was used to inform the number of CSTs. Bray-Curtis dissimilarity scores were calculated between consecutive paired specimens from each participant. The heatmap was generated using the ComplexHeatmap package^65^ and the same metrics used to identify CSTs. Change of CST was defined as change or no change in CST between consecutive paired specimens.

### Statistical analysis

Statistical models that accounted for repeated measures within individuals were fitted using generalised estimating equations (GEE) to investigate the impact of characteristics and practices on the diversity (Shannon-Diversity Index) and instability (change of CST or Bray-Curtis dissimilarity) of the vaginal microbiota. GEE linear regression analyses were used when the outcome was Shannon-Diversity Index or Bray-Curtis dissimilarity, with the regression coefficient representing the mean difference of each outcome between the reference and comparison group/s for each characteristic/practice investigated. GEE logistic regression was used when change of CST was the outcome. Characteristics and practices deemed significant in univariate analyses (p < 0.05) were included in multivariable analyses.

We also analysed the type of CST change observed. Specimens were allocated one of four change type between sequential specimens: (1) no change; (2) change from one *Lactobacillus* CST to another *Lactobacillus* CST; (3) change from one *Lactobacillus* CST to a non-*Lactobacillus* CST; or (4) change from a non-*Lactobacillus* CST to any other CST. Multinomial regression was used to investigate the relationship between practices and type of CST change relative to the risk of no change, generating relative risk ratios and 95% confidence intervals.

Multinomial regression was also used to assess associations between characteristics and microbiota composition (i.e. CST-classification of a sample). CST1-*L. crispatus* was the reference group for all analyses. This analysis calculated the risk of having a vaginal microbiota of a specific CST (details of CSTs provided in results below) compared to the risk of a vaginal microbiota of CST1, clustering for multiple samples from individual participants.

Characteristics and practices deemed significant in univariate analyses (p < 0.05) were included in multivariable analyses. Statistical analyses were performed using STATA v14.2, unless otherwise specified.

## Supplementary information

Supplementary information

## Data Availability

The raw sequencing data are publicly available in the NCBI Sequence Read Archive (SRA) under the Bioproject number PRJNA434520.
