# Supplementary material for: Sexual practices have a significant impact on the vaginal microbiota of women who have sex with women
Source: Sci Rep. 2019 Dec 24;9:19749. doi: 10.1038/s41598-019-55929-7 (PMC6930205; doi:10.1038/s41598-019-55929-7)
Supplement: Supplementary file 1 — Supplementary information [file 41598_2019_55929_MOESM1_ESM.docx]

**Supplementary information**

**Sexual practices have a significant impact on the vaginal microbiota of women who have sex with women**

Erica L. Plummer^1,2,a*^, Lenka A. Vodstrcil^1,2,a^, Christopher K. Fairley^1,2,^, Sepehr N. Tabrizi^3,4,5^, Suzanne M. Garland^3,4,5^, Matthew G. Law^6^, Jane S. Hocking^7^, Katherine A. Fethers^2^, Dieter M. Bulach^8,9^, Gerald L. Murray^3,4,5,b^, Catriona S. Bradshaw^1,2,7,b^

^1^Central Clinical School, Monash University, The Alfred Centre, Melbourne, Victoria

^2^Melbourne Sexual Health Centre, Alfred Health, Carlton, Victoria

^3^Women’s Centre for Infectious Diseases, The Royal Women’s Hospital, Parkville, Victoria

^4^ Murdoch Children’s Research Institute, Melbourne, Victoria

^5^ Department of Obstetrics and Gynaecology, The University of Melbourne, Parkville, Victoria

^6^ Kirby Institute, UNSW Australia, Kensington, NSW

^7^Melbourne School of Population and Global Health, The University of Melbourne, Parkville, Victoria

^8^ Microbiological Diagnostic Unit Public Health Laboratory, The Peter Doherty Institute for Infection and Immunity, The University of Melbourne, Melbourne, Victoria

^9^ Melbourne Bioinformatics, The University of Melbourne, Carlton, Victoria

^a^ Erica L. Plummer and Lenka A. Vodstrcil contributed equally to this manuscript.

^b^ Gerald L. Murray and Catriona S. Bradshaw contributed equally to this manuscript.

**Supplementary table 1 – Effect of new sexual partner exposure on microbiota diversity, stratified by oral sex**

|  |  | **Diversity ceoff. (95% CI)** ^a^ | **p-value** |
| --- | --- | --- | --- |
| **No oral sex**  **(n=138)** | No new partner (n=124) | ref |  |
|  | New partner (n=14) | 0.263 (-0.07,0.60) | 0.127 |
| **Oral sex**  **(n=221)** | No new partner (n=175) | ref |  |
|  | New partner (n=46) | 0.517 (0.27,0.76) | **<0.001** |

^a^ Univariate GEE linear regression, where participant ID is panel variable. The regression coefficient represents the mean difference of Shannon diversity between the reference (i.e. no new sexual partner) and comparison group (i.e. new sexual partner).

**Supplementary Table 2 – Characteristics and practices associated with vaginal microbiota composition by univariate multinomial logistic regression**

| **Characteristic** | **CST2**  ***Lactobacillus* mixed** | | **CST3**  ***L. iners*** | | **CST4**  ***G. vaginalis* and diverse** | | **CST5**  **Anaerobic and diverse** | |
| --- | --- | --- | --- | --- | --- | --- | --- | --- |
|  | (N=44) ^a^ | | (N=93) ^a^ | | (N=40) ^a^ | | (N=31) ^a^ | |
|  | RRR (95% CI) | *P value* | RRR (95% CI) | *P value* | RRR (95% CI) | *P value* | RRR (95% CI) | *P value* |
| Self-reported past history of BV |  | | | | | | | |
| No | 1 |  | 1 |  | 1 |  | 1 |  |
| Yes | 0.87 (0.35,2.17) | 0.762 | 0.69 (0.26,1.87) | 0.467 | 1.13 (0.39,3.27) | 0.817 | 2.82 (1.09,2.27) | **0.032** |
| ***Longitudinal practices^b^*** | | | | | | | | |
| Any smoking^c^ |  | | | | | | | |
| No | 1 |  | 1 |  | 1 |  | 1 |  |
| Yes | 1.59 (0.77,3.29) | 0.214 | 1.38 (0.71,2.65) | 0.341 | 1.72 (0.74,4.01) | 0.207 | 3.01 (1.31,6.92) | **0.009** |
| Any douching^d^ |  |  |  |  |  |  |  |  |
| No | 1 |  | 1 |  |  |  | 1 |  |
| Yes | 1.73 (0.15,20.04) | 0.660 | 2.48 (0.58,10.69) | 0.222 | … | … | 5.14 (1.20,21.96) | **0.027** |
| Onset of last menses |  |  |  |  |  |  |  |  |
| > 7 days ago | 1 |  | 1 |  | 1 |  | 1 |  |
| ≤ 7 days ago | 3.59 (1.49,8.68) | **0.004** | 2.19 (1.08,4.46) | **0.030** | 1.64 (0.55,4.91) | 0.378 | 1.03 (0.27,4.00) | 0.964 |
| Number of SP |  |  |  |  |  |  |  |  |
| 0 | 1 |  | 1 |  | 1 |  | 1 |  |
| 1 | 0.60 (0.22,1.68) | 0.334 | 0.68 (0.20,2.27) | 0.528 | 0.72 (0.18,2.85) | 0.639 | 0.45 (0.13,1.61) | 0.222 |
| ≥2 | 0.51 (0.12,2.25) | 0.374 | 0.83 (0.20,3.40) | 0.800 | 1.72 (0.36,8.26) | 0.498 | 1.91 (0.48,7.65) | 0.360 |
| Frequency of sex |  | | | | | | | |
| Once/month or less | 1 |  | 1 |  | 1 |  | 1 |  |
| Several times/month | 1.15 (0.46,2.90) | 0.763 | 0.79 (0.39,1.61) | 0.513 | 0.73 (0.27,2.00) | 0.543 | 1.51 (0.59,3.85) | 0.386 |
| Several times/week | 1.62 (0.64,4.07) | 0.306 | 1 (0.45,2.21) | 1.000 | 1.49 (0.52,4.33) | 0.459 | 1.37 (0.47,3.93) | 0.563 |
| Sex with NP^e^ |  | | | | | | | |
| No | 1 |  | 1 |  | 1 |  | 1 |  |
| Yes | 0.62 (0.18,2.19) | 0.460 | 1.77 (0.87,3.60) | 0.117 | 4.09 (1.69,9.92) | **0.002** | 5.37 (2.18,13.20) | **<0.001** |
| ***Sexual practices with FSP*** | | | | | | | | |
| Any receptive oral vaginal sex |  | | | | | | | |
| No^f^ | 1 |  | 1 |  | 1 |  | 1 |  |
| Yes | 1.09 (0.56,2.15) | 0.799 | 1.09 (0.57,2.12) | 0.790 | 2.60 (1.09,6.20) | **0.031** | 2.17 (0.86,5.46) | 0.099 |
| Sharing of sex toys with FSP | | | | | | | | |
| No toys/washed/condoms used^f^ | 1 |  | 1 |  | 1 |  | 1 |  |
| Unwashed | 0.92 (0.36,2.32) | 0.859 | 0.98 (0.50,1.92) | 0.952 | 2.38 (1.04,5.45) | **0.039** | 1.46 (0.62,3.46) | 0.387 |
| ***Sexual practices with MSP*** | | | | | | | | |
| Any vaginal sex |  | | | | | | | |
| No^g^ | 1 |  | 1 |  | 1 |  | 1 |  |
| Yes | 0.61 (0.11,3.32) | 0.564 | 2.64 (0.94,7.44) | 0.066 | 1.41 (0.29,6.82) | 0.666 | 2.45 (0.64,9.30) | 0.189 |
| Any receptive oral vaginal sex |  | | | | | | | |
| No^g^ | 1 |  | 1 |  | 1 |  | 1 |  |
| Yes | 0.98 (0.15,6.41) | 0.983 | 3.96 (1.05,14.92) | **0.042** | 1.67 (0.24,11.60) | 0.605 | 3.96 (0.76,20.59) | 0.102 |
| Any anal sex |  | | | | | | | |
| No^g^ | 1 |  | 1 |  | 1 |  | 1 |  |
| Yes | 3.49 (0.22,54.60) | 0.373 | 12.21 (1.25,119.66) | **0.032** | 3.85 (0.24,60.51) | 0.338 | 10.34 (0.91,117.70) | 0.060 |
| ***Self-reported symptoms*** | | | | | | | | |
| Self-reported abnormal vaginal discharge and/or odour |  | | | | | | | |
| No | 1 |  | 1 |  | 1 |  | 1 |  |
| Yes | 0.23 (0.03,1.78) | 0.159 | 1.06 (0.44,2.56) | 0.904 | 3.29 (1.21,8.91) | **0.019** | 4.03 (1.54,10.59) | **0.005** |

Abbreviations: BV, bacterial vaginosis; SP, sexual partner (may refer to FSP or MSP); NP, new partner (may refer to FSP or MSP); FSP, female sexual partner; MSP, male sexual partner

Missing data for variables included in this analysis occurred in <0.5% of intervals.

^a^ Multinomial logistic regression with CST1-*L. crispatus* as the baseline comparison group (n=152). Analysis clustered for multiple specimens from participants (100 clusters).

^b^ Longitudinal characteristics were measured as any exposure over the prior follow-up interval (~90 days). No significant associations were identified between microbiota composition and hormonal contraceptive use.

^c^ There was no dose-response relationship between smoking and Shannon diversity

^d^ Douching was rarely practice, as such it was not included in adjusted analyses.

^e^ Sex with a new partner with who first sexual contact was within 90 days

^f^ Or did not have a FSP

^g^ Or did not have a MSP
